# Intrinsic capacity and health-promoting lifestyle in older adults: a latent class analysis

**DOI:** 10.3389/fpubh.2025.1634373

**Published:** 2025-07-16

**Authors:** Chuyun Xu, Jiaying Yu, Lili Yang, Yu Li, Dongchi Ma

**Affiliations:** School of Nursing, Zhejiang Chinese Medical University, Hangzhou, Zhejiang, China

**Keywords:** intrinsic capacity, older adults, healthy aging, health-promoting lifestyles, latent class analysis

## Abstract

**Objectives:**

This study aims to explore the latent classes and characteristics of older adults’ intrinsic capacity and health-promoting lifestyle, and to investigate the associations between intrinsic capacity and categories of health-promoting lifestyle.

**Methods:**

A total of 800 older adults from five communities in Hangzhou, Zhejiang Province, were recruited using a convenience sampling method. Data were collected through the administration of a general information questionnaire, the ICOPE simple screening tool, and the Health Promotion Lifestyle Scale. Latent class analysis is employed to investigate the correlation between intrinsic capacity and health-promoting lifestyle among older adults. Additionally, multinomial logistic regression is utilized to explore the influencing factors associated with different latent classes.

**Results:**

The intrinsic capacity and health-promoting lifestyle among older adults can be categorized into three latent classes: low psychological-low health type (15%), relatively healthy type (60%), and low cognitive-low participation type (25%). Pearson correlation analysis revealed a significant correlation between intrinsic capacity scores and health-promoting lifestyle scores (*r* = 0.357, *p* < 0.001). Age, educational level, number of comorbidities, history of falls within the past 3 months, and sleep duration were identified as significant influencing factors for the latent classes of intrinsic capacity and health-promoting lifestyle among older adults (all *p* < 0.05).

**Conclusion:**

Individuals aged 60 to 79 years, with higher educational levels, ≤2 chronic diseases, no history of falls in the past 3months, and more than 6 h of sleep per day are more likely to be categorized into the relatively healthy type. In contrast, those with ≥3 chronic diseases and less than 6 h of sleep per day are more likely to be classified into the low psychological–low health type.

## Introduction

The global population is experiencing significant aging, with the number of people aged 60 and over expected to exceed 2 billion by 2050 (1). To actively promote healthy aging, the World Health Organization (WHO) proposed a new concept of intrinsic capacity (IC) in 2015 (2), which refers to the composite of all physical and mental capacities of an individual including six dimensions of locomotion, cognition, vitality, vision, hearing, and psychology (3). It essentially determines whether older adults can achieve healthy aging (4) and is influenced by age, gender, disease status, chronic inflammation, lifestyle, and social environment, etc. (5). Health-promoting lifestyle (HPL) is a series of health behaviors taken by individuals to improve their physical and mental health, including cognition, emotion, and activities (6). Research has indicated that taking a health-promoting lifestyle among older adults can substantially decrease the risk of age-related illnesses and disabilities. Existing studies have made efforts to categorize IC levels and explore their influencing factors (7, 8). The integration of these two concepts is complementary at the theoretical level, providing a comprehensive framework for healthy aging by supporting the goal from both the “capacity reserve” and “behavioral practice” perspectives. This approach can be used to construct a multidimensional framework for healthy aging. Latent class analysis (LCA) is a population-centered classification technique that can effectively identify groups of people with common characteristics (9). This study aims to apply LCA to form latent classes of intrinsic capacity and health-promoting lifestyles for older adults, and will analyze the characteristics of these classes and their influencing factors, which can offer insights for healthcare professionals to develop personalized integrated care plans for older adults, promoting healthy aging.

## Materials and methods

### Participants

From August 2024 to March 2025, the researchers adopted convenient sampling method to select older adults who were treated in outpatient clinics of five community health service centers in Hangzhou, Zhejiang Province for this study. Inclusion criteria: (1) Aged≥60 years; (2) Voluntarily participated and could comprehend the questionnaire items. Exclusion criterion: People with severe dementia or other mental disorders. In a report on the application of latent class analysis to health (10), a sample size of 500 is considered valuable in most simulation studies. In this study, the sample size was estimated using the formula *N* = [*Z*_2*α*/2_ (1-*p*) *p*]/θ^2^, with *α* = 0.05 and *Z*_α/2_ = 1.96. Given the 43.0% decline rate of IC in Chinese community-dwelling older adults (*p* = 0.43) (11) and a permissible error (*δ*) of 3%, the calculated sample size was approximately 534. Considering a 10% invalid questionnaire rate, the final required sample size was set at a minimum of 588. This study has passed the ethics committee’s expedited review (No. 20240712–6). All participants were fully informed and signed consent forms.

### Survey tool

#### General information questionnaire

General information mainly includes sociodemographic data and other relevant data. The questionnaire content is as follows: (1) Sociodemographic data: gender, age, height, weight, place of residence, living status, marital status, educational level, type of health insurance, and monthly income. (2) Other data: chronic diseases, polypharmacy (≥5 medication use) (yes/no), sleep duration, recent falls (within 3 months) (yes/no).

#### ICOPE screening tool (integrated care for older people)

The ICOPE screening tool consists of a preliminary screening and a basic assessment scale, both of which can be used to screen for IC in older adults. In this study, the preliminary screening scale recommended by the WHO was adopted (12). The cognitive domain includes three items: time and place orientation tests, immediate memory tests, and recall tests from the Minimum Mental State Examination (MMSE). The physical domain uses the five repetition sit-to-stand test from the Short Physical Performance Battery (SPPB). The vitality (nutrition) domain uses two Mini-Nutritional Assessment (MNA) items related to weight and appetite loss. The vision domain involves asking about hyperopia, myopia, or other eye diseases. The hearing domain involves asking about hearing impairment. The psychological domain includes two Patient Health Questionnaire (PHQ-9) items related to emotional problems. One point will be awarded for each domain that performs well, and 0 point otherwise. The final score ranges from 0 to 6. A higher score indicates better IC in older adults (13–15).

#### Health promoting lifestyle profile-II (HPLP-II)

The Chinese version of HPLP-II was developed in 2016 (16), which has been widely used among older adults, adolescents, and chronic disease patients. When applied to community residents, it has a Cronbach’s α coefficient of 0.93 and a split-half reliability of 0.89. This scale includes six dimensions: interpersonal relations, nutrition, health responsibility, physical exercise, stress management, and spiritual growth, with a total of 40 items. It uses a Likert four-point scoring method (1 = never, 2 = sometimes, 3 = often, 4 = always), with total scores ranging from 40 to 160. Higher scores indicate better health-promoting lifestyle behaviors.

### Data collection

Investigators were trained to ensure data accuracy and consistency before the survey, and were required to use a unified guidance to explain the study’s purpose, significance, and confidentiality principles to participants, and answered any questions that arose during questionnaire completion. After the survey, we promptly checked the completeness and validity of the data, verified and corrected any questionable entries, and excluded data with errors that could influence the results. A total of 855 questionnaires were distributed, all of which were collected on the spot, with 800 valid ones recovered, yielding an effective response rate of 93.6%.

### Data analysis

Data analysis was performed using SPSS26.0 and Mplus8.0. Since the scores of health-promoting lifestyle items are not dichotomous, a formula was applied: score index = (actual score/full score) × 100%. A score index ≤60% was coded as “0,” and >60% as “1,” converting item scores into dichotomous variables. Count data were described with frequency and percentage, and measurement data with mean ± standard deviation (M ± SD). The significance level was set at *p* < 0.05. The Pearson correlation test was employed to examine the correlation between intrinsic capacity scores and health-promoting lifestyle scores. We used Mplus 8.0 to conduct latent class analysis on the study subjects. The number of classes in the model was incrementally increased from one, and the optimal model was determined based on a comprehensive evaluation of fit indices, including Akaike Information Criterion (AIC), Bayesian Information Criterion (BIC), Sample Size-adjusted BIC (aBIC), Entropy, Lo–Mendell–Rubin Likelihood Ratio Test (LMR), and Bootstrapped Likelihood Ratio Test, (BLRT). Chi-square tests were employed to examine differences in demographic characteristics across latent classes through univariate analysis. Subsequently, multinomial logistic regression analysis was performed with latent class membership as the dependent variable.

## Results

### Demographic characteristics

Of the 800 participants, 387 (48.4%) were male with a mean age of 73.1 years, and 413 (51.6%) were female with a mean age of 73.0 years. 213 (26.6%) had more than three conditions (this is referred to as multimorbidity in the following text, Individuals suffering from three or more chronic diseases simultaneously represent a more complex and severe form of multimorbidity. In this study, the number of diseases is set at three as the boundary to explore the relationship between the number of chronic diseases and latent classes), 212 (26.5%) had polypharmacy (≥5 medication use), and 210 (26.3%) had an education level below primary school. 189 (23.6%) had a monthly income below 3,000, 305 (38.1%) slept less than 6 h per day, and 439 (54.9%) previously engaged in light-physical-labor jobs. Based on the latent class model, participants were classified into three distinct classes. Significant differences were found across classes in age, gender, chronic disease, medication use, educational level, monthly income, health insurance type, sleep duration, occupational background, and recent falls (within 3 months) (all *p*-values <0.05). Details are in Table 1.

**Table 1:** Comparison of general data of different latent classes.

Items	Class 1	Class 2	Class 3	*χ*^2^	*P*
Gender					
Male	49 (41.9)	227 (47.0)	111 (55.5)	6.408	0.041
Female	68 (58.1)	256 (53.0)	89 (44.5)		
Age					
60 ~ 69	29 (24.8)	182 (37.8)	48 (24.0)		
70 ~ 79	56 (47.9)	235 (48.8)	93 (46.5)	33.734	<0.001
≥80	32 (27.4)	65 (13.5)	59 (29.5)		
Chronic disease					
<3	65 (55.6)	375 (77.6)	147 (73.5)	23.515	<0.001
≥3	52 (44.4)	108 (22.4)	53 (26.5)		
≥5 medication use					
Yes	50 (42.7)	105 (21,7)	57 (28.5)	21.864	<0.001
No	67 (57.3)	378 (78.3)	143 (71.5)		
Educational level					
Primary school and below	45 (38.5)	84 (17.4)	81 (40.5)	49.57	<0.001
Junior high school and higher	72 (61.5)	399 (82.6)	119 (59.5)		
Monthly income					
≤3,000	37 (31.6)	97 (20.1)	55 (27.5)		
3,001 ~ 5,000	49 (41.9)	181 (37.5)	71 (35.5)	14.6	0.024
5,001 ~ 10,000	30 (25.6)	192 (39.8)	70 (35.0)		
≥10,000	1 (0.9)	13 (2.7)	4 (2.0)		
Sleep duration					
<6 h	71 (60.7)	153 (31.7)	81 (40.5)	34.231	<0.001
>6 h	46 (39.3)	330 (68.3)	119 (59.5)		
Occupation					
Light physical labor	47 (40.2)	296 (61.3)	96 (48.0)		
Moderate physical labor	37 (31.6)	104 (21.5)	50 (25.0)	23.639	<0.001
Heavy physical labor	33 (28.2)	83 (17.2)	54 (27.0)		
Health insurance					
Urban medical insurance	90 (76.9)	434 (89.9)	164 (82.0)	16.626	<0.001
Rural health care coverage	27 (23.1)	49 (10.1)	36 (18.0)		
Recent falls (within 3 months)					
Yes	21 (17.9)	38 (7.9)	19 (9.5)	10.897	0.004
No	96 (82.1)	445 (92.1)	181 (90.5)		

### Fit and classification of latent class model

**Figure 1:**
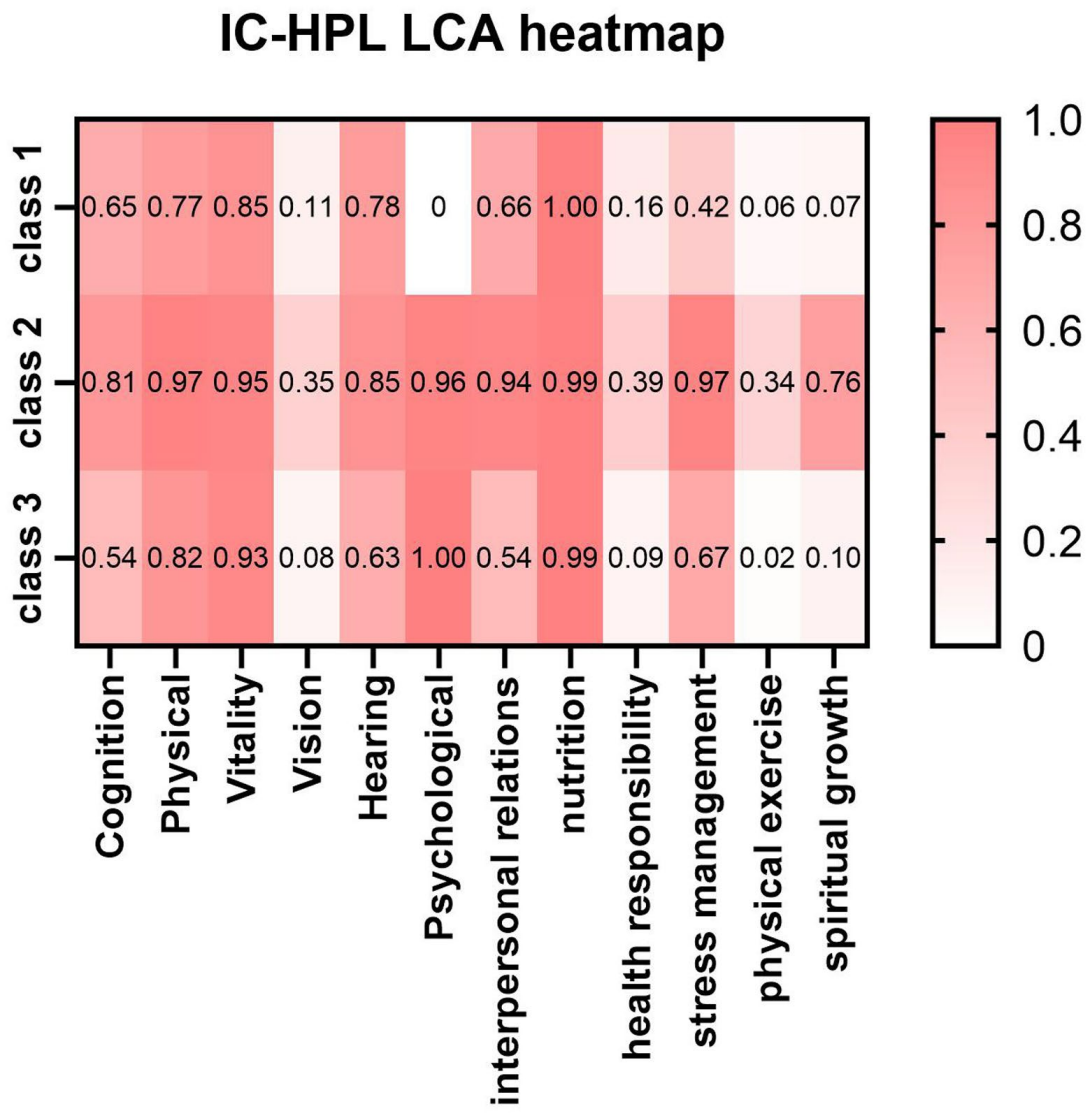
IC-HPL latent class analysis heatmap.

As shown in Table 2, this study explored four latent class models. As the number of classes increased, AIC, BIC, and aBIC generally showed a downward trend. The LMR and BLRT values were significant when the number of classes was 2 and 3 (all *P* < 0.001). However, the Entropy value was greater than 0.8 only when the number of classes was 3, with a value of 0.812. The Entropy value ranges from 0 to 1. A value closer to 1 indicates clearer classification. A value above 0.8 suggests the model fits the data well and its classification is acceptable. Moreover, both LMR and BLRT values were less than 0.001, indicating a high classification accuracy and a good model fit. Taking all factors into account, the final decision was made to select three classes as the outcome of the latent class analysis of intrinsic capacity and health-promoting lifestyle among older adults (Model 3). Figure 1 illustrates the performance of three classes of older adults in terms of “intrinsic capacity” and “health-promoting lifestyle.” Darker shading represents higher performance levels within each dimension. The scoring trends of the three classes of older adults in terms of intrinsic capacity and health-promoting lifestyle are depicted in Figure 1. Individuals in class 1 accounted for 15% of the sample. They had significantly lower scores across all dimensions, with the lowest scores in the psychological, stress management, and spiritual growth dimensions. This suggests that these individuals may experience mental health issues and face the dual challenges of poor stress management and inadequate spiritual growth. Therefore, this class was labeled as the “low psychological-low health type.” Class 2 accounted for 60% of the sample. It was characterized by relatively good performance in all domains except for three distinct low scores in vision, health responsibility, and physical activity. These low scores were not unique to class 2, as all three classes exhibited this trend. Therefore, class 2 was named the “relatively healthy type.” Class 3, comprising 25% of the sample, had the lowest scores among the three classes in cognition, interpersonal relationships, health responsibility, and physical activity. Consequently, this class was labeled as the “low cognition-low participation type.”

**Table 2:** Adaptability test indexes of latent class models.

Model	AIC	BIC	aBIC	Entropy	*P*		Class probability
					LMR	BLRT	
1	8928.974	8985.189	8947.082	/	/	/	1
2	8404.397	8521.512	8442.123	0.698	<0.001^*^	<0.001^*^	0.61/0.39
3	8382.235	8560.250	8439.579	0.812^*^	<0.001^*^	<0.001^*^	0.15/0.60/0.25
4	8374.185	8613.101	8451.147	0.673	0.1181	<0.001^*^	0.31/0.26/0.14/0.29

AIC, Akaike Information Criterion; BIC, Bayesian Information Criterion; aBIC, sample size-adjusted BIC; LMR, Lo–Mendell–Rubin Likelihood Ratio Test; BLRT, Bootstrapped Likelihood Ratio Test.

*Statistical significance.

### The distribution of health-promoting lifestyle scores and IC

The health-promoting lifestyle scores of all older adults are presented in Table 3. The total scores of the Health-Promoting Lifestyle scale for older adults ranged from a minimum of 65 to a maximum of 137, with a mean score of 102.21. Given the inconsistent total scores across different dimensions, the raw scores of each dimension were first converted to *Z*-scores to facilitate interdimensional comparison. Subsequently, the *T*-score conversion was performed using the formula: T = 50 + 10Z. The T-score converted results for each dimension among the older adult in different classes are presented in Table 4. Specifically, individuals classified as the “low psychological-low health type” and the “low cognition-low participation type” exhibited lower scores across all dimensions of the Health-Promoting Lifestyle Scale. In contrast, those categorized as the “relatively healthy type” achieved the highest scores. Among these classes, the low psychological-low health type had the lowest scores in the stress management and spiritual growth dimensions compared to the other two classes. The relatively healthy type had generally higher scores across all dimensions, with only the health responsibility and physical exercise dimensions scoring relatively lower, although still higher than the other two types. The low cognitive-low participation type had the lowest scores in the interpersonal relations, nutrition, health responsibility, and physical exercise dimensions among the three classes. Table 5 presents the performance of older adults in each dimension of IC. The highest proportions of good performance were observed in the physical and vitality dimensions, with 720 (90%) and 744 (93%) individuals, respectively. Psychological function followed, with 664 individuals (83%). Hearing and cognition came next, with 622 (77.8%) and 570 (71.3%), respectively. The lowest proportion of good performance was in vision, with only 194 individuals (24.3%). As shown in Table 6, the total IC scores of older adults ranged from 1 to 6. The majority of individuals (67.4%) had scores between 4 and 5. The proportion of individuals with no decline in IC was 12.9%. Pearson correlation analysis revealed a positive correlation between IC scores and health-promoting lifestyle among older adults (*r* = 0.357, *p* < 0.001).

**Table 3:** The scores of the health-promoting lifestyle for older adults ( $\bar{x}$ ± s).

Dimensions	Minimum value	Maximum value	Mean ± standard deviation
Interpersonal relations	8	20	14.58 ± 2.497
Nutrition	17	31	25.07 ± 2.356
Health responsibility	9	29	16.87 ± 3.268
Stress management	8	23	16.23 ± 2.388
Physical exercise	9	30	16.5 ± 3.148
Spiritual growth	7	27	12.66 ± 2.328
HPLP-II scores	65	137	102.21 ± 10.872

HPLP-II, Health Promoting Lifestyle Profile-II.

**Table 4:** The scores of health-promoting lifestyles for older adults in different latent classes ( $\bar{x}$ ± s).

Dimensions	Low psychological – low health type	Relatively healthy type	Low cognition – low participation type
Interpersonal relations	46.244 ± 0.889	54.042 ± 0.360	42.435 ± 0.673
Nutrition	47.656 ± 0.934	51.734 ± 0.429	47.183 ± 0.735
Health responsibility	47.132 ± 0.798	53.031 ± 0.446	44.358 ± 0.575
Stress management	41.303 ± 0.828	54.449 ± 0.344	44.343 ± 0.678
Physical exercise	45.200 ± 0.693	53.573 ± 0.458	44.179 ± 0,474
Spiritual growth	41.428 ± 0.539	55.078 ± 0.409	42.752 ± 0.367

**Table 5:** The proportion of older adults with good performance in various dimensions of IC.

Dimensions	Cognition	Physical	Vitality	Vision	Hearing	Psychological
Number (%)	570 (71.3)	720 (90.0)	744 (93.0)	194 (24.3)	622 (77.8)	664 (83.0)

**Table 6:** The distribution of the total IC scores of older adults.

IC scores	1	2	3	4	5	6
Number (%)	4 (0.5)	27 (3.4)	127 (15.9)	238 (29.8)	301 (37.6)	103 (12.9)

### Multinomial logistic regression analysis for older adults

Collinearity diagnostics showed that all independent variables had VIFs below 5 and tolerance values above 0.2, indicating no significant multicollinearity. Thus, no variables need to be removed and all can be included in the multinomial logistic regression. Before conducting the regression analysis, dummy variables were created for occupation and health insurance type, while no dummy variables were created for other binary and ordinal categorical variables. Taking class 1 (low psychological–low health type) as the reference class, the final statistical analysis results (Table 7) showed that the model was significant overall (*χ*^2^ = 166.431, *p* < 0.001). Individuals aged 60 ~ 79 years, with higher education levels, no multimorbidity, no history of falls in the past 3 months, and more than 6 h of sleep per day were more likely to be classified as relatively healthy type. Those with multimorbidity and less than 6 h of sleep per day were more likely to be classified as low psychological-low health type.

**Table 7:** Multiple logistic regression of different latent classes.

Factors	Class 2			Class 3		
	OR	95%CI	*P*	OR	95%CI	*P*
Age						
60 ~ 69	2.776	(1.447,5.327)	0.002	0.832	(0.418,1.655)	0.600
70 ~ 79	2.581	(1.456,4.575)	0.001	1.014	(0.568,1.812)	0.962
≥80	/	/	/	/	/	/
Sleep duration						
<6 h	0.271	(0.171,0.430)	<0.001	0.494	(0.302,0.809)	0.005
>6 h	/	/	/	/	/	/
Educational level						
Primary school and lower	0.509	(0.277,0.932)	0.029	1.709	(0.902,3.240)	0.100
Junior high school and higher	/	/	/	/	/	/
Chronic disease						
<3	2.203	(1.234,3.932)	0.008	2.056	(1.085,3.899)	0.027
≥3	/	/	/	/	/	/
Recent falls						
Yes	0.469	(0.246,0.893)	0.021	0.540	(0.267,1.093)	0.087
No	/	/	/	/	/	/

## Discussion

### General

The results indicated that the three latent classes shared commonalities as well as differences. In terms of health-promoting lifestyles, all three latent classes in this study scored high in nutrition but low in health responsibility and physical exercise. This pattern aligns with previous research (17), this may be due to a combination of factors, such as insufficient perceived health capacity and social support, limitations in the environment and facilities, and suboptimal physical health (18–20). Research has highlighted that perceived health ability and exposure to health education significantly influence health-promoting behaviors in older adults (21). To address this, it’s crucial to enhance their perceived health ability. Communities can organize diverse health education activities, such as health lectures, sharing videos and articles on WeChat public accounts, hosting health knowledge competitions, forming community health promotion groups, and conducting volunteer services. These initiatives can improve older adults’ health literacy and self-efficacy, promote exchanging and sharing among them, and thus boost their level of health responsibility. Studies have shown that exercise plays a vital role in the prevention of numerous chronic diseases, such as cardiovascular diseases, stroke, diabetes, osteoporosis, and obesity. Moreover, exercise can also improve frailty, enhance psychological well-being, and boost the quality of life (22). Therefore, for all three latent classes, personalized exercise plans should be developed, and exercise programs can be adjusted in terms of volume and intensity based on the health levels and medical conditions of different classes of older adults.

### The characteristics of three latent classes analysis

The results indicated that there were significant differences among the three classes in terms of psychological dimensions and spiritual growth, while the scoring trends in other dimensions were consistent.

#### Class 1

Class 2 and 3 had similar scores in the psychological dimension, whereas class 1 had significantly lower scores in this dimension. This may have been related to the weaker stress management capabilities and poorer psychological resilience of older adults in class 1, which made them more prone to psychological health problems (23, 24). It is evident that there is a need to enhance the emotional regulation and stress management capabilities of older adults in class 1 in order to improve their psychological health levels. Social support is a crucial factor in alleviating stress among older adults. Studies have shown that older adults with a strong social support network are more likely to receive emotional comfort and support, thereby mitigating the negative impacts of stress (25). In addition, mindfulness-based therapy, cognitive-behavioral therapy, and rational emotive behavior therapy have all been shown to significantly mitigate the impact of stress on the psychological health of older adults (26). The results of the multinomial logistic regression analysis indicated that older adults with multimorbidity and sleep duration of less than 6 h were more likely to be classified as the low psychological-low health type. This suggests that the burden of chronic diseases and sleep issues may exacerbate psychological distress and diminish adaptive capacity among older adults, which is consistent with previous research (27, 28). For older adults with these characteristics, intervention measures should start from enhancing their sense of control over health and introduce chronic disease self-management programs. Moreover, sleep issues often co-occur with depression and chronic pain. Studies have showed that decreased sleep duration and poor sleep quality are linked to cognitive decline and depression (29, 30). Additionally, extreme sleep duration (≤4 h or ≥10 h) is identified as a risk factor for cognitive decline (29). Therefore, it is essential to improve the lifestyles of older adults with extreme sleep duration. This can be achieved by encouraging regular exercise, participation in social activities, and maintaining consistent daily routines, which can help promote sleep quality and healthy aging (31, 32). Sleep and mood integrated interventions are also applicable to this class of older adults. Digital cognitive behavioral therapy for insomnia (CBT-I) has been proven to simultaneously improve sleep quality and psychological resilience in older adults (33).

#### Class 2

Class 2 showed relatively low scores in vision, health responsibility, and physical activity, though still higher than the other two classes. Given their satisfactory performance in other dimensions, these older adults exhibit relatively good physical and mental health overall. However, their visual impairment, as well as their weaker health awareness and physical activity levels, may pose long-term risks, warranting further attention. Visual impairment is prevalent among older adults, research has showed that older adults with visual impairment tend to have lower levels of social participation and interaction compared to those with good vision (34). This highlights the regulatory role of maintaining good vision in promoting healthy lifestyle choices among older adults, particularly in dimensions closely linked to social participation and interaction, such as interpersonal relationships and physical exercise. Given that social interaction is a key determinant of healthy aging (35), communities should enhance vision screening and care for older adults, raise their awareness about vision health to prevent the adverse effects of visual impairment on cognitive function and social functioning. Multinomial logistic regression analysis revealed that older adults aged 60 ~ 79 years, without multimorbidity, with higher education levels, sleeping more than 6 h, and no history of falls in the past 3 months were more likely to be classified into this type. Their relatively healthy physiological status may contribute to a “health optimism bias”—where positive self-perceptions of health lead to underestimating health risks—resulting in lower health responsibility scores and neglect of the benefits of physical activity. For this specific subgroup, intervention strategies could be developed by leveraging the peer effect (where individuals’ behaviors are influenced by their social peers) and applying principles from social support theory (which emphasizes the role of interpersonal relationships in promoting health behaviors). Such approaches may help counteract their over-optimistic self-assessment and encourage greater engagement in preventive health measures (36, 37). Peer mentoring and support programs have been demonstrated as evidence-based interventions. Trained teams comprising younger health professionals and experienced older volunteers can co-design and implement age-appropriate exercise programs, thereby enhancing participation rates and optimizing health outcomes. Furthermore, peer support networks leverage modeling effects and social accountability to sustain long-term exercise adherence, effectively mitigating motivation-related dropout among older populations (36, 38). Group dynamics theory can also be utilized to promote healthy behaviors among older adults. Group cohesion can enhance the health behaviors of older adults through the spirit of mutual assistance and cooperation and the norms of reciprocity. For instance, organizing group activities or mutual aid groups for older adults can strengthen their health awareness and thereby promote proactive medical-seeking behaviors (39).

#### Class 3

Class 3 has the lowest scores in the dimensions of cognition, vision, hearing, interpersonal relations, health responsibility, and physical exercise among the three classes. Since low scores in vision, health responsibility, and physical exercise are common characteristics of all three classes, which have been discussed in the preceding text, we will only focus on the unique attributes of class 3 here. First, older adults in class 3 are characterized by declines in both cognitive and auditory functions. The Sensory Deprivation Hypothesis posits that long-term lack of sufficient sensory input (such as hearing or vision loss) can lead to neuronal atrophy, which in turn triggers cognitive decline (40, 41). Research has indicated that hearing interventions, such as the use of hearing aids, may play a positive role in delaying cognitive decline (42). This suggests that interventions for older adults in class 3 should prioritize hearing rehabilitation. Additionally, older adults in class 3 exhibit low levels of interpersonal relations. Research has indicated that hearing loss is correlated with decreased social participation among older adults (43), and cognitive decline further limits their social capabilities. This dual impairment creates a vicious cycle of social isolation: sensory and cognitive deficits lead to reduced social interaction, which in turn exacerbates cognitive decline (44, 45). For this population, combined hearing and cognitive interventions, enhanced intergenerational interactions, and the use of digital technology to assist in improving social participation and cognitive levels can be implemented (46–48).

### Limitations

This study still has several limitations. First, the nature of this study is cross-sectional, which means that it captures data at a single point in time, it cannot establish definitive causality between variables but can only suggest potential risk factors or directional influences. For example, if we find a correlation between HPL scores and IC scores, we cannot conclude that the lower HPL scores directly causes the lower IC scores based on this study alone. It can only provide insights into potential risk factors or directional influences. That is, it can suggest that one variable may be associated with another in a particular direction, but further research, such as longitudinal studies or experimental designs, would be needed to confirm causality. Second, the use of convenience sampling may limit sample representativeness, however, multi-center sampling partially mitigated this issue. By collecting data from multiple centers, the sample diversity was enhanced. This means that participants from different locations, with potentially varying characteristics, were included. But it is still possible that there are certain characteristics or subgroups of the population that are underrepresented or not represented at all in the sample. Third, the assessment of IC in vision and hearing relied on self-reported data, which may introduce measurement bias. Self-reported data is subjective and can be influenced by various factors such as the participants’ memory, perception, and honesty. For instance, individuals may overestimate or underestimate their abilities due to social desirability bias or inaccurate self-assessment. This can introduce measurement bias, which may affect the accuracy and reliability of the results related to IC in vision and hearing. Forth, the process of converting continuous variables into dichotomous variables may lead to information loss and narrow the scope. Using 60% as the cut-off point is the result of following the conventions in our research field. The choice of this cut-off point was not made through other methods, which is a limitation of this study. Additionally, the study focused on community-dwelling older adults who had relatively preserved IC. This limits the insights that can be gained regarding health-promoting behaviors among those who have experienced significant IC decline. Older adults with substantial IC decline may have different health-promoting needs and behaviors compared to those with preserved IC. By including nursing home residents in future research, a more diverse range of sample characteristics can be obtained. Lastly, the specific characteristics of the local population, such as cultural background, lifestyle, and healthcare system, may influence the results. Therefore, the generalizability of the findings to other regions may be limited. Conducting similar studies in other regions with different demographic and environmental factors could enhance the applicability of the results and provide a more comprehensive understanding of the phenomenon being studied.

## Conclusion

Latent class analysis revealed that there is heterogeneity in the intrinsic capacity–health-promoting lifestyle among older adults, which can be divided into three latent classes: low psychological-low health type (15%), relatively healthy type (60%), and low cognitive-low participation type (25%). Older adults aged 60 ~ 79 years, with higher education levels, ≤2 chronic diseases, no history of falls in the past 3 months, and more than 6 h of sleep per day are more likely to be classified as the relatively healthy type. Those with ≥3 chronic diseases and less than 6 h of sleep per day are more likely to be classified as the low psychological-low health type. Interventions for class 1 should focus on chronic disease management, improving sleep quality, and enhancing stress management and psychological resilience. For class 2, the emphasis should be on maintaining health, increasing health responsibility, and strengthening physical exercise. For class 3, interventions should focus on enhancing cognitive and hearing levels, improving interpersonal relationships, and promoting social participation.

## Data Availability

The raw data supporting the conclusions of this article will be made available by the authors, without undue reservation.
